# Orienting attention to semantic categories

**DOI:** 10.1016/j.neuroimage.2006.08.017

**Published:** 2006-12

**Authors:** Tamara C. Cristescu, Joseph T. Devlin, Anna C. Nobre

**Affiliations:** a University of Oxford, Department of Experimental Psychology, South Parks Road, Oxford, OX1 3UD, UK; b Centre for Functional Magnetic Resonance Imaging of the Brain, University of Oxford, UK

**Keywords:** Spatial attention, Left inferior frontal cortex, Parietal cortex

## Abstract

We investigated the ability to orient attention to a complex, non-perceptual attribute of stimuli—semantic category. Behavioral consequences and neural correlates of semantic orienting were revealed and compared with those of spatial orienting, using event-related functional magnetic-resonance imaging. Semantic orienting significantly shortened response times to identify word stimuli, showing that it is possible to focus attention on non-perceptual attributes of stimuli to enhance behavioral performance. Semantic-orienting cues engaged parietal and frontal areas that were also involved in spatial orienting, but in addition engaged brain areas associated with semantic analysis of words, such as the left anterior inferior frontal cortex. These findings show that attentional orienting selectively engages brain areas with functional specialization for the predicted attributes. They also support the existence of a core frontoparietal network, which controls attentional orienting in speeded response tasks independently of the type of expectations, interacting with task-relevant functionally specialized areas to optimize perception and action.

## Introduction

Adapting flexibly to the ever-changing environmental stimulation and demands is crucial to human behavior. The ability to alter preparatory states and focus resources selectively on task-relevant information in order to optimize behavioral performance is known as attentional orienting (Posner, 1980; Posner et al., 1980). Traditionally, research into attentional orienting mechanisms has concentrated on the ability to focus resources on simple perceptual attributes of stimuli. Specifically, most research has investigated spatial (Posner, 1980) or object-based attention (Duncan, 1984). Recent investigations have established that attention improves performance in a wider range of tasks than originally thought. For example, attention can be deployed to non-perceptual attributes of stimuli such as their predicted motor responses (Rushworth et al., 2003) or temporal intervals (Griffin and Nobre, 2005; Nobre, 2001b).

Brain-imaging and neuropsychological studies have shown that attentional orienting relies on sensorimotor frontoparietal circuits. Spatial orienting is structured around a right-hemisphere dominant network including areas around the intraparietal sulcus and the frontal eye fields (FEF) (Gitelman et al., 1999; Mesulam, 1981, 1999; Nobre et al., 1997; Weintraub et al., 1996), which overlaps with the network for oculomotor control (Corbetta, 1998; Nobre et al., 2000). The network for object-based attention shares neural substrates and mechanisms with that for spatial orienting (Nobre, 2001a; Serences et al., 2004; Wojciulik and Kanwisher, 1999). Orienting attention to motor responses relies on a left-dominant network including supramarginal gyrus and inferior premotor areas, overlapping with circuits for control of manual responses (Rushworth et al., 2001, 2003). Orienting attention to instants in time engages brain areas involved in spatial as well as motor orienting (Coull and Nobre, 1998; Nobre, 2001b).

Two principles thus emerge: (1) Attentional orienting functions are flexible and able to operate on different types of information to optimize behaviors; and (2) frontoparietal circuits involved in sensorimotor integration provide the core system for attentional orienting functions (Nobre, 2004). However, one important limit to the studies to date precludes strong conclusions. Studies so far have manipulated attention to stimulus attributes linked to perceptual analysis (locations, objects and features), motor responses, or a combination of these (temporal instants). The frontoparietal sensorimotor circuits supporting attentional orienting could therefore be determined by the sensory/motor nature of the information relevant to orienting in these tasks.

The current study tests the flexibility of attentional orienting and the ubiquity of frontoparietal control systems by investigating the ability to orient attention to abstract associative features of stimuli. Specifically, we tested the ability to orient attention to semantic categories of words. Early behavioral studies have suggested that it is possible to build semantic expectations based upon probabilistic relations between semantic categories, which facilitate recognition of word stimuli (Neely, 1977; Posner and Snyder, 1975a). There is also behavioral evidence that semantic associations between concrete stimuli can influence deployment of spatial attention, biasing attention toward associated stimuli in a visual search task (Moores et al., 2003). To our knowledge, however, there are no studies that have investigated the brain areas supporting semantic orienting of attention.

**Fig. 1:**
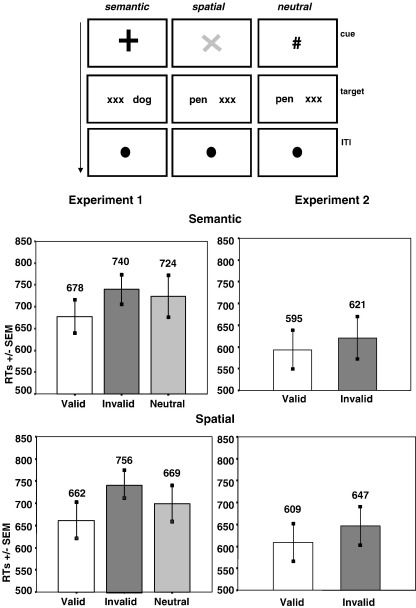
Experimental task schematic and behavioral results in Experiments 1 and 2. The cueing stimulus (Experiment 1: ×, +, #; Experiment 2: ×, +) was presented at the beginning of each trial. The cue stayed on the screen during the whole cue-target interval. Targets then appeared in the left or right VF, accompanied by a string of ×'s of the same length in the opposite VF. A fixation point was presented at the end of each trial to help participants refocus on the center of the screen. Experiment 2 did not have a neutral cueing condition. Graphs show behavioral performance (mean of median RTs and standard error) in the semantic and spatial attention conditions for Experiment 1 and Experiment 2.

We designed two event-related fMRI experiments to investigate the behavioral and neural effects of semantic orienting and its relation to spatial orienting. Both experiments used a cued lexical-decision task with a similar structure to the Posner attentional orienting task (Posner, 1980) (Fig. 1). Verbal (word or non-word) stimuli were presented visually at peripheral locations, preceded by symbolic cues carrying predictive semantic or spatial information. Semantic cues predicted the most likely semantic category of the target word (animal or tool), and spatial cues predicted the most likely location of the target words (left or right visual field). Behavioral responses as well as the brain systems supporting both forms of orienting were compared in two the experiments.

The first experiment was specifically designed to determine whether orienting attention to the semantic category of a word would facilitate behavioral performance in a similar fashion to orienting to its spatial location. Predictive cues were expected to induce specific expectations about upcoming word targets designating animals or tools and presented in the left or right VF. Valid cues provided correct semantic or spatial information about the target stimulus and helped participants deploy attention efficiently. We expected an improvement in behavioral performance induced by valid cues relative to invalid or neutral cues. In addition, this experiment further investigated the neural effects of semantic and spatial orienting associated with each type of cue. Here, there were two possible alternatives. Our working hypothesis was that the neural basis of attentional orienting would be dictated by the type of expectation afforded by predictive information. In this case, left-hemisphere brain areas involved in semantic analysis of linguistic stimuli (Vandenberghe et al., 1996) would be involved, such as inferior prefrontal areas around pars triangularis and pars orbitalis (Devlin et al., 2003; Gitelman et al., 2005; Gough et al., 2005) and anterior medial temporal cortex (Nobre and McCarthy, 1995; Nobre et al., 1994). The alternative possibility was that semantic orienting would be fully supported by the right-hemisphere dominant frontoparietal network supporting attentional orienting to perceptual attributes (Corbetta and Shulman, 2002; Gitelman et al., 1999; Hopfinger et al., 2000). This result would support the hypothesis that an all-purpose, general cortical network sustains different forms of attentional orienting regardless of the specific content of expectations demanded by the task.

The second experiment was designed to validate the cueing results from the first experiment, which included a limited number of participants. The second experiment used a simplified design, which maximized the number of predictive semantic and spatial cues, while maintaining the duration of the task tolerable for participants. This second experiment also used a larger proportion of valid-to-invalid semantic and spatial trials compared to the previous experiment.

## Methods and procedures

### Participants

Eight participants (aged between 20 and 27 years, 4 females) participated in Experiment 1, and a separate set of 12 participants (aged 19–31 years, 10 females) participated in Experiment 2. All were right-handed (Oldfield, 1971) native English speakers with normal or corrected-to-normal vision and none reported any history of neurological disease or taking any medication. Participants gave informed written consent. The study was approved by the Central Oxford Research Ethics Committee.

Unfortunately, hardware problems with the scanner limited the number of participants in both studies. The first experiment was truncated early due to a scanner hardware failure and subsequent update, which interrupted data acquisition and prevented the continuation of the study. In the second experiment, imaging data were collected from 12 participants but instability of the gradients compromised data quality in four of these participants, leaving only eight participants in the fMRI analysis. Further acquisitions were prevented by subsequent scanner upgrades, which addressed these gradient problems. Consequently, special care was taken with the data analyses to draw conservative inferences (see below).

### Task and stimuli

Participants performed a cued lexical-decision task (Fig. 1). In each trial, a cue appeared centrally for a variable duration, followed by a briefly presented target array (100 ms duration). Trials were separated by a variable inter-trial interval (ITI). Target arrays consisted of two letter-strings presented peripherally, each centered at 5° eccentricity along the horizontal meridian. One string was either a word or a pronounceable non-word (pseudoword), while the other was a string of Xs of the same length in the complementary location of the opposite visual field. Participants responded as rapidly as possible using a right index-finger button press when a real word was present in either location. In Experiment 1, the cue duration and ITI varied between 2 and 16 s, with intervals skewed toward shorter durations (mean = 7 s) to decrease overall length of the experiment. In Experiment 2, cue duration and ITI varied between 2 and 6 s (mean = 4 s).

In the first experiment, targets were preceded by predictive or neutral symbolic cues. Predictive cues enabled participants to develop specific types of expectations about the upcoming target. There were two types of predictive cues: semantic and spatial. Semantic cues predicted the likely semantic category of an upcoming word target (animal or tool). Spatial cues predicted the location of an upcoming word target (left or right visual field). The majority of predictive cues were valid. Neutral cues did not provide any predictive information about the semantic category or spatial location of upcoming target words. The symbols “×” and “+” served as both semantic and spatial cues across subjects with the color of the cue (red or green) designating the type of information predicted. Assignment of the specific symbol and color were fully counterbalanced among participants. The “#” symbol always designated a neutral cue and it could appear in either color. The main difference in design between the first and second experiment was that neutral cues were not included in the second experiment.

Experiment 1 consisted of 168 trials. There were 60 trials with semantic cues (30 animals, 30 tools), 60 trials with spatial cues (left, right), and 48 trials with neutral cues. In all conditions, cues were followed by words in 2/3 of the trials and by pseudowords on 1/3 of the trials. Words were equally likely to come from the two semantic categories (animals, tools), and appeared equally likely in the two visual fields (left, right). Semantic cues were followed by words in 40 trials, and by pseudowords in 20 trials. Within the word trials, 80% of the cues were valid (32 trials) and 20% of the cues were invalid (8 trials). Trial types after spatial cues followed the same pattern: 40 word trials and 20 pseudoword trials; with 80% of word trials containing valid cues (32) and 20% containing invalid cues (8). Neutral cues were followed by words in 32 trials and by pseudowords in 16 trials.

Trials with semantic, spatial, and neutral cues were intermixed in a randomized order in a purely event-related design. Event onsets were “jittered” to avoid any systematic sampling bias and to avoid temporal expectations (Griffin and Nobre, 2005). Participants completed two runs of 84 trials, each lasting about 20 min. The tasks were prepared and presented using Presentation® software (Version 0.50, http://www.neurobs.com).

The second experiment contained 160 trials, all of which had predictive cues. There were 80 semantic cues and 80 spatial cues. In each condition, cues were followed by word targets in 90% of trials, and by pseudowords in the remaining 10% of trials. Words were equally divided between the two semantic categories and the two visual fields. Semantic and spatial cues were each followed by words in 72 trials and by pseudowords in 8 trials. Within the word trials, 89% of the cues were valid (64 trials) and 11% were invalid (8 trials).

Stimuli were presented in a mixed block and event-related design. Trials were clustered into alternating blocks containing only semantic cues or only spatial cues. Before each block, an instruction frame (2 s duration) indicated the type of block to follow. Five trials were presented in each block, lasting on average of 42 s. The active blocks were separated by 15 s of rest, during which the participant maintained fixation on a central stimulus. Within active blocks, trial types were ordered pseudo-randomly and were unpredictable. The order in which the semantic and spatial blocks were presented (ABAB… or BABA…) was counterbalanced over participants.

In both experiments, the real words used were short concrete nouns (3–8 letters) taken from the MRC Psycholinguistic Database (Coltheart, 1981) (Table 1). Half of the concrete nouns represented animals and half manipulable tools. They were matched for frequency, familiarity, and number of letters. Pseudowords were matched for string length and were constructed by merging the initial segment of an animal word with the end of a tool word or vice versa.

Both experiments were piloted before scanning. For the first experiment, a separate group of eight subjects was used to ensure that the task could be performed in the absence of eye movements and with a high level of accuracy. For the second, the same participants used in the main experiment took part in a behavioral pilot session 1 to 5 days prior to scanning to ensure that they understood the task and that they were able to perform the task without eye movements. A separate version of the task, using different stimuli was prepared for this purpose (120 trials). An infrared eye-tracker (iView 3.0, SensoMotoric Instruments GmbH) was used to monitor eye movements in both pilot experiments. All subjects were able to maintain fixation on the central cue without any systematic eye movements.

### Behavioral analysis

Behavioral analyses probed for benefits in speed and accuracy of responses induced by valid semantic or spatial orienting. Only participants with good accuracy of performance were considered in the behavioral analyses. Outliers were defined as responses occurring two standard deviations above or below the participant's average RT. Outlying values were removed before the RT and accuracy analysis.

The overall performance in the lexical-decision tasks was assessed with analyses of variance (ANOVAs), which tested the effects of cue validity on RTs to word targets. The factors tested were cue type: semantic and spatial; and cue validity: valid, invalid (and neutral in the first experiment). Responses were collapsed over factors of secondary interest, which were balanced in the design: target type (animal, tool) and VF of presentation (left, right VF) to ensure a stable measure of accuracy across all conditions. Trial numbers, especially in the invalid-cueing condition, would have been too low otherwise.

### Image acquisition

During the fMRI session, participants lay supine in the scanner bed with the right hand on the button box. Tilted mirrors were positioned over the eyes, so they could view the screen in front of the scanner onto which the stimuli were projected (Sanyo PLC-XP40L, 1024 × 768-pixel resolution). Foam pads were placed around the participants' heads in order to minimize movements. Earplugs and MR-compatible headphones were used to attenuate scanner noise. Participants were asked to maintain central visual fixation during the duration of the experimental task, and to read target stimuli using peripheral vision only. During the experiment, the light was turned off in the scanner room to reduce distraction.

Functional and anatomical images were acquired with a Varian-Siemens 3 T scanner at the Centre for Functional Magnetic Resonance Imaging of the Brain in Oxford. A Magnex head-dedicated gradient insert coil was used in conjunction with a birdcage head radio frequency coil tuned to 127.4 MHz. Functional imaging consisted of 24 T2*-weighted echo-planar image (EPI) slices (TR = 3 s, TE = 30 ms, FOV = 192 × 256 mm, matrix = 64 × 64, flip angle 90°) giving a notional voxel resolution of 3 × 4 × 5 mm. An automated shimming algorithm was used to reduce magnetic field inhomogeneities (Wilson et al., 2002).

In Experiment 1, approximately 400 volumes were acquired during each run lasting around 20 min each. These covered the entire cortical surface, but sampling of the cerebellum was incomplete in some cases. Each functional run began with 12 s during which instructions were presented to remind participants of the cue assignments and to allow for T1 magnetic equilibrium. Scans acquired in this interval were discarded before analysis. In Experiment 2, functional images were acquired during one single run per subject, consisting of approximately 590 sets of axial slices, and lasting approximately 30 min. During the run, blocks of trials with semantic and spatial cues alternated 16 times. The order of tasks was counterbalanced across subjects.

In addition, T1-weighted scans were acquired (3D Turbo FLASH sequence, TR = 15 ms, TE = 6.9 ms) with 1 mm^2^ in-plane resolution and 1.5 mm slice thickness for the purpose of registration and anatomical localization in both experiments.

### Image analysis

Data were processed and analyzed using Statistical Parametric Mapping (SPM2, Wellcome Department of Cognitive Neurology, London UK) running on MATLAB (MathWorks Inc., USA). Images were realigned and unwarped to reduce non-linear distortions due to magnetic field inhomogeneities (Andersson et al., 2001). Motion reports showed that, in both experiments, none of the participants moved more than 3 mm during an imaging session. Structural scans were spatially coregistered with the realigned functional images to enable anatomical localization of the activations. All images were spatially normalized into a standardized anatomical framework using the averaged-brain template of the Montreal Neurological Institute (Collins et al., 1994). Functional images were spatially smoothed with an 8-mm Gaussian filter. The time series were temporally filtered to remove sources of slow drift (high-pass filter: 128 s). The statistical analysis employed a general linear model (Friston et al., 1994, 1995). Task events were modeled using the canonical hemodynamic response function (Friston et al., 1998; Glover, 1999). Temporal derivatives were also included as covariates of no interest to improve statistical sensitivity by removing regional deviations in timing from the canonical HRF.

For the first experiment, activity correlated with each type of cue was estimated, including semantic cues predicting animals, semantic cues predicting tools, spatial cues predicting left, spatial cues predicting right, and neutral cues. In addition, we modeled activity correlated with target arrays. Different types of target arrays were combined into a single explanatory variable. Statistical comparisons between experimental factors used linear contrasts in a fixed-effects analysis. The small number of participants precluded effective use of a random-effects model and therefore the reliability of the findings was tested by their replication across subjects and in a separate experiment. In order to ensure that the findings were consistent over subjects, only brain areas whose activation was significant in 75% or more of the subjects (≥ 6) were considered reliable. This stringent procedure ensured between-subject reliability at some potential risk of missing real areas of activation (i.e. Type II error). Inclusive masking was used to identify regions that were commonly activated by both semantic and spatial cues. Activations evoked by semantic cues relative to the implicit baseline periods (i.e. fixation) were masked by those evoked by spatial cues relative to the implicit baseline (*p* < .05 uncorrected). Brain areas preferentially engaged by semantic versus spatial cues (and vice-versa) were identified by linear contrasts contrasting the activations in each case [(semantic minus spatial); (spatial minus semantic)].

In the second experiment, the model similarly included conditions for each type of cue (left, right, animals, tools) and one condition for targets. The short cue-target intervals were not well suited for revealing common activations for the semantic and spatial cues, but could individuate areas activated differentially by semantic or spatial cues. In order to test the reliability of the results in Experiment 1, we tested for significant results using the same linear contrasts within a 10-mm radius of activations in Experiment 1, which was within the spatial resolution of the data sets (≥ 12 mm FWHM). In both analyses, activations were considered significant using a voxel-wise statistical test at *p* < .05 after correcting for multiple comparisons at the family-wise level (Friston et al., 1994; Worsley et al., 1996).

Following the group-level analyses, the magnitude of the activations (parameter estimates) in the brain regions showing differential responses for semantic versus spatial cues was extracted and plotted, to show the pattern of brain activations and the nature of their modulation across conditions. In both experiments, spherical regions with 8 mm radius around the peak activation were defined using the MarsBar region-of-interest toolbox (Brett et al., 2002). Parameter estimates were extracted from the regions of greater activations elicited by semantic cues: left inferior frontal gyrus, left posterior middle temporal gyrus, angular gyrus (Table 2, see below). Parameter estimates were extracted for each subject and averaged separately for the semantic and spatial cueing conditions.

As a further test of the reliability of the results obtained with the two separate fixed-effects analyses, a between-studies random-effects analysis was also conducted. The analysis focused on areas differentially activated by semantic cues relative to spatial cues, and included the relevant contrast (semantic minus spatial cues) evaluated separately for each participant in the first and the second experiment.

All results were rendered on a high-resolution structural image of a single subject's brain in MNI space. The MRIcro software was used to create the figures (http://www.cla.sc.edu/psy/faculty/rorden/mricro.html). Activations are presented in neurological convention (left = left).

## Results

### Behavioral effects of semantic and spatial orienting to words

The behavioral analysis included all eight participants in the first experiment. In the second experiment, one participant was excluded from the behavioral analysis due to technical problems with recording her behavioral performance during the scanning session. Therefore, the final behavioral analysis included 11 participants in the second experiment.

The accuracy measures in both experiments demonstrated that participants could perform the tasks adequately and that performance was not at ceiling. All participants detected target nouns with high accuracy in the first experiment (78% correctly identified target words in the semantic condition and 78% correctly identified target words in the spatial condition). Accuracy of detecting word targets was also high in the second experiment (81% correctly identified target words in the semantic condition and 73% correctly identified target words in the spatial condition). Accuracy of detecting pseudowords was high in both experiments (82% in the first experiment and 91% in the second experiment).

**Fig. 2:**
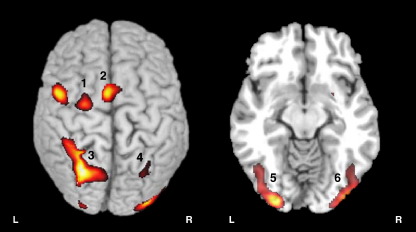
Brain areas activated in common by semantic and spatial orienting cues. Semantic and spatial cues commonly activated left frontal areas in lateral premotor cortex including FEF (1) and pre-SMA (2); posterior parietal areas in left intraparietal sulcus and superior parietal lobule (3) and right intraparietal sulcus (4); and visual cortex (5 and 6). Activations are shown superimposed on a representative brain volume normalized to the standardized brain (MRICro) in this and subsequent figures, using neurological convention (left = left).

Reaction times (RTs) for each of the experimental conditions are shown in Fig. 2. The RT analysis revealed a main effect of cue validity [*F*_(2,14)_ = 6.71, *p* = .009]. Follow-up pairwise comparisons showed that validly cued words elicited significantly shorter RTs (670 ms) than invalidly cued words (748 ms) [*F*_(1,7)_ = 19.61, *p* = .003]. RTs to neutrally cued words were intermediate (712 ms) and did not differ significantly from RTs to validly or invalidly cued words. There was no main effect of cue type, suggesting similar performance in the semantic and spatial orienting conditions in the first experiment (Fig. 2).

Experiment 2 replicated the semantic- and spatial-orienting effects of the previous experiment (Fig. 1). The RT analysis revealed a significant effect of cue validity [*F*_(1,10)_ = 5.36, *p* = .043] as validly cued targets elicited shorter RTs (601 ms) than invalidly cued targets (633 ms). There was no main effect of cue type, meaning that similar cue-validity effects were presented in the semantic- and spatial-orienting conditions (Table 2).

### Brain areas activated by semantic and spatial orienting to words

The imaging data from the first experiment identified the set of brain areas engaged by orienting cues. We began by determining which regions were commonly engaged by semantic and spatial orienting. In this experiment, variable cue durations and variable inter-stimulus intervals (see Methods and procedures) enabled us to compare and contrast activation specific to orienting attention toward either semantic categories or spatial locations based on the cueing stimuli. Several brain areas were commonly activated by both types of cues (Table 2 and Fig. 2). Activation was prominent in posterior parietal cortex, around the intraparietal sulcus and superior parietal lobule. Frontal cortex was activated in medial and lateral premotor and posterior prefrontal areas, including the frontal eye fields. Parietal and frontal activations were more extensive in the left hemisphere than in the right hemisphere. Orienting cues also elicited bilateral visual activation extending from the lateral occipital gyri to ventral occipitotemporal areas, and was continuous with activation in the cerebellum.

### Brain areas selectively activated by semantic cueing

**Fig. 3:**
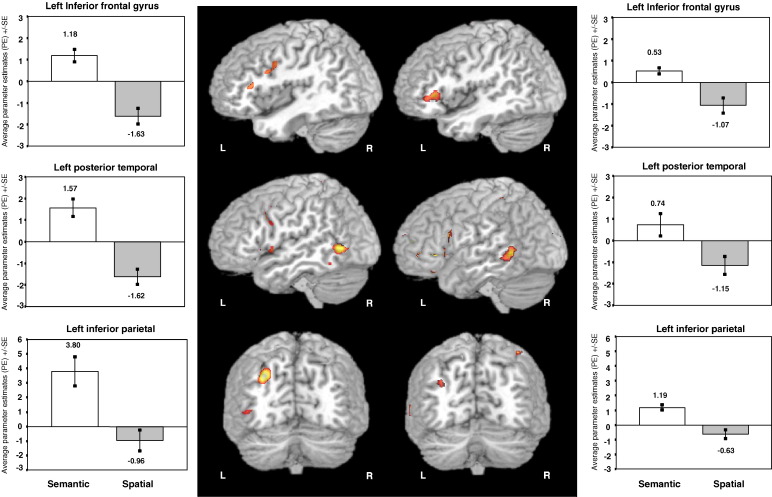
Brain areas selectively activated by semantic cues in Experiment 1 (left) and Experiment 2 (right). For each region, the mean signal change, separated according to cue type (semantic, spatial, or neutral), and standard errors are plotted adjacent to the relevant regions of interest.

We identified brain areas that were preferentially engaged by orienting to semantic categories versus spatial locations by directly contrasting semantic and spatial cues. Semantic relative to spatial cues activated a reliable set of left-hemisphere brain areas (Table 3 and Fig. 3). Frontal activations were observed in the left inferior frontal cortex (LIFC) and lateral inferior premotor cortex. Additional activations were observed in the left posterior temporal cortex around the middle temporal gyrus and inferior temporal sulcus, and in the left angular gyrus of the inferior parietal lobule. Most of these foci were selectively activated in the semantic orienting condition. One exception was the lateral inferior premotor cortex, which was commonly activated by both types of expectations (see Table 3), but was more activated in the semantic orienting condition. No brain area was more activated for spatial cues relative to semantic cues.

The second experiment replicated these effects of semantic relative to spatial cueing (Table 3 and Fig. 3). Left inferior frontal cortex, left lateral premotor cortex, posterior temporal cortex, and inferior parietal in the angular gyrus were also preferentially activated by semantic cues. In addition, the second experiment showed selective activation in the left anterior medial temporal cortex during semantic orienting. Like the previous experiment, the comparison of spatial relative to semantic cues did not reveal any significant areas of activation.

An additional between-studies random-effects analysis replicated the pattern of preferential activations for semantic compared to spatial cues observed in the two separate fixed-effects analyses of both experiments. Semantic cues were associated with higher activations than spatial cues in left LIFC (peak coordinate: − 45 36 6), inferior premotor cortex (− 45 15 30), left posterior temporal cortex (− 54 − 60 3), and left inferior parietal cortex (− 36 − 66 30) (*p* < .05, corrected). No activation was observed in the anterior medial temporal cortex.

Plots of the effect sizes in the activated regions showed that the greater activation in these areas by semantic cues compared to spatial cues resulted from differences in positive activations, and showed the areas to be significantly activated by semantic cues relative to the implicit baseline (Fig. 3). Activity in the anterior medial temporal cortex observed in the second experiment was also higher for semantic compared to spatial cues, but activity to semantic cues was not reliably higher than during the implicit baseline. Unfortunately, because of the high degree of signal drop-out and distortion in the echo-planar images in this region (Devlin et al., 2000), it may not have been possible to obtain reliable measurements from this brain area.

## Discussion

The aims of this study were to investigate whether it is possible to orient attention to semantic categories and to identify the neural system supporting this form of attentional orienting in the human brain. The results support and extend the emerging notion of attentional orienting as a flexible set of cognitive functions that can operate at a variety of levels of representation to enhance behavioral performance, and advance our understanding of the neural bases of these functions.

The behavioral results showed that attention can be oriented to abstract features such as semantic representations to improve behavioral efficiency, confirming early behavioral demonstrations of semantic orienting (Neely, 1977; Posner and Snyder, 1975a,b). Behavioral performance was enhanced by both semantic and spatial orienting to a similar extent in the experiments. Of particular interest for our study was the facilitatory effect of valid semantic cueing, which demonstrated that semantic cues induced valid expectations that guided attention to semantic categories of upcoming words.

The behavioral advantage enjoyed by validly cued stimuli is due to voluntary attentional orienting to semantic information. Semantic expectations were driven by symbolic cues, which did not contain intrinsic semantic information that could be automatically associated with the target word. This greatly limits the possibility that the spreading of automatic semantic associations (Collins and Loftus, 1975; Meyer and Schvaneveldt, 1971) between the cue and target stimuli might have affected the behavioral results. Semantic orienting is therefore different from semantic priming, though both may involve developing expectations and the use of semantic matching processes (Neely, 1991) to influence lexical decisions.

Both experiments used a high prevalence of word trials, and of valid trials, which is likely to have accentuated semantic expectations developed by the cues. On the other hand, the low proportions of pseudowords are unlikely to have differentially engaged semantic-matching processes, which become accentuated at high non-word proportions (Neely et al., 1989). Furthermore, any effects of semantic matching would be restricted to the target period, and not the cue-related activations, which were the focus of the fMRI analysis.

The brain-imaging results showed that semantic orienting in the current tasks engaged frontoparietal networks, which were also activated by spatial orienting. In addition, semantic orienting also selectively engaged activation in left-hemisphere brain areas known to participate in semantic analysis of word stimuli. These findings therefore support the interpretation that brain areas supporting attentional orienting are at least partly determined by the type of predictive information (Nobre, 2004). However, the findings are also consistent with the existence of a core set of brain areas that support attentional orienting in speeded response tasks more generally, independently of the type of expectations involved.

### Selective brain areas for semantic orienting

Semantic cues in both experiments selectively activated brain areas participating in semantic analysis of word stimuli, specifically left IFG around pars triangularis, angular gyrus, and posterior middle temporal gyrus (Price, 2000). These activations were triggered by the presentation of semantic cues, which appeared before any word targets were presented. Therefore, we can conclude that they do not reflect semantic processing of words per se, but rather attentional orienting to the semantic category of the upcoming word target. One cannot rule out the possibility that the cue stimuli engaged semantic processing for the retrieval of their associated meaning, but this would not have differed between semantic and spatial cues.

Research so far suggests that the left posterior ventrolateral prefrontal and premotor cortex, the region broadly labeled as Broca’s region, is a complex structure containing subregions with different functional specializations relevant to language (Gabrieli et al., 1998). The more anterior region within pars triangularis and pars orbitalis has been shown to be activated in tasks emphasizing semantic analysis of word stimuli. This region shows preferential activation for semantic versus phonological processing, and interference with brain activity in this region selectively disrupts semantic processing (Devlin et al., 2003; Gitelman et al., 2005; Gough et al., 2005). Although no consensus has been reached about the exact role of LIFC, most interpretations suggest a role in top-down processes such as selection and retrieval of semantic information (Cardillo et al., 2004; Gold and Buckner, 2002; Thompson-Schill et al., 1997; Wagner et al., 2001). Our results are consistent with a top-down role of the LIFC related to semantic analysis, and show that it may be involved in generating and/or maintaining semantic expectations about the semantic category of the upcoming word target, which facilitate subsequent processing of word stimuli.

Semantic cues also selectively activated angular gyrus and posterior temporal cortex. These two regions have been associated with accessing or processing semantic information in single-word or sentence reading tasks (Bavelier and Corina, 1997; Price et al., 1996; Vandenberghe et al., 1996). In addition, intracranial recordings, brain imaging, and neuropsychological observations have also implicated the anterior medial temporal cortex in semantic analysis (Hodges et al., 1992; Mummery et al., 1999, 2000; Nobre and McCarthy, 1995; Nobre et al., 1994; Rossell et al., 2003). Stronger activation was observed in this region for semantic cues compared to spatial cues only in the second experiment. Unfortunately, BOLD signal in this last region of the brain is notoriously poor, which may have compromised the ability to observe reliable modulation of this brain region.

We propose that these brain areas represent key nodes in a widely distributed network integrating and retrieving semantic knowledge. The multimodal nature of this circuit enables the formation of selective semantic expectations and the biasing of brain activity by these semantic expectations.

### Common brain areas for attentional orienting

Semantic and spatial orienting also activated a number of parietal and frontal brain regions in common. The frontoparietal pattern of activation was characteristic of visuospatial attention orienting tasks in terms of activated areas (Corbetta and Shulman, 2002; Hopfinger et al., 2000; Yantis and Serences, 2003). In our experiment, the activations in this network were strongly left-lateralized, differing somewhat from some studies showing more numerous or more extensive dorsal posterior parietal activations over the right hemisphere (e.g., Corbetta et al., 1993; Gitelman et al., 1999) and the common finding of bilateral activations of the dorsal parietal and frontal areas during the anticipatory control of spatial orienting.

Activation of the frontoparietal network by semantic orienting cues supports the existence of a ubiquitous and general purpose attentional orienting network. The multimodal network for spatial cognition and attention may provide a useful system for coordinating different types of expectations about events and to influence multiple levels of stimulus analysis. After all, events exist within spatial frameworks. However, it is also possible that the activation of frontoparietal areas in our task was not specifically linked to semantic orienting. Instead, it could represent the inadvertent contribution of spatial factors in the semantic orienting conditions. Words were presented at one of two possible spatial locations within the visual field, which probably induced spatial expectations about the target location even in the semantic orienting condition. Follow-up studies of semantic orienting using foveal stimulus presentations may help constrain the interpretation about frontoparietal involvement. However, even foveal stimuli exist within a spatial framework and could in principle engage spatial expectations. Indeed, frontoparietal areas have been activated in tasks of attentional orienting toward non-spatial factors even when foveal stimuli have been used (e.g., Coull et al., 2000). This apparently simple potential confound, may therefore prove difficult to eliminate.

Our finding of a strong left-hemisphere bias in the frontoparietal network raises the interesting possibility that the hemispheric contribution to this network may be influenced by the task context. In the current task, even spatial orienting showed left lateralization. This may have resulted from the linguistic context in our task. The need for the frontoparietal network to interact with language-related brain areas in the task may have biased activity toward the left hemisphere. We are currently testing this possibility with follow-up experiments.

## Figures and Tables

**Table 1:** Psycholinguistic parameters of word stimuli (animals and tools) used in the experimental task

	Experiment 1		Experiment 2	
Animal	Tool	Animal	Tool
Letters (3–8)	5.1 (0.2)	4.8 (0.2)	5.1 (0.2)	5.3 (0.2)
KF-frequency	14 (3.0)	9 (1.9)	6 (0.7)	7 (0.7)
Familiarity	500 (10)	481 (9)	474 (7)	592 (7)
Concreteness	574 (8)	608 (4)	600 (4)	608 (4)

Psycholinguistic parameters of word stimuli—means (standard errors).

**Table 2:** Brain areas activated by semantic and spatial orienting to words

Brain Area	Peak voxel (*x y z* mm)	*Z*-score	Subjects (*N*)
*Frontal*			
L lateral premotor: PCS/IFS	− 50 + 06 + 40	> 8.00	6
L lateral premotor/prefrontal: FEF	− 26 − 04 + 62	> 8.00	7
L medial premotor: pre-SMA	− 06 + 06 + 60	> 8.00	6

*Parietal*			
L posterior parietal: IPS and SPL	− 28 − 64 + 50	> 8.00	8
− 30 − 50 + 48	> 8.00	6
R posterior parietal: IPS and SPL	+ 28 − 68 + 50	6.97	6

*Occipital and cerebellum*			
L occipital: lateral occipital gyrus	− 30 − 92 − 08	> 8.00	8
L occipital: post fusiform/occipitotemporal sulcus	− 42 − 80 − 14	6.77	6
L cerebellum	− 38 − 60 − 26	5.68	8
R occipital: lateral occipital gyrus	+ 32 − 92 − 04	> 8.00	7
R occipital: occipitotemporal sulcus	+ 43 − 68 − 16	6.80	6
R cerebellum	+ 38 − 64 − 32	6.78	7

Common activations during semantic orienting and spatial orienting. The standard space coordinates of the peak voxel are shown in millimeters along with the corresponding *Z*-score at that point. The number of subjects (*N*) showing this activation at *p* < .01 (uncorrected) is shown in the final column.

**Table 3:** Brain areas selectively activated by semantic cueing

Brain area	Experiment 1			Experiment 2		
Peak (mm)	*Z*-score	*N*	Peak (mm)	*Z*-score	*N*
*Frontal*						
L inferior frontal: PTr	− 46 + 28 + 14	4.66	7	− 48 + 30 + 03	5.41	7
			− 45 + 42 + 00	5.45	7
L lateral premotor:PCS/IFS	− 44 + 04 + 38	4.64	6	*− 33 + 03 + 54*	*4.02*	*7*

*Temporal*						
L post temporal: MTG/ITS	− 54 − 62 + 00	4.83	6	− 66 − 48 + 00	4.66	7
L anterior medial temporal	–			− 18 − 12 − 30	4.88	6

*Parietal*						
L inferior parietal: AG	− 32 − 72 + 38	5.38	6	*− 36 − 69 + 30*	*3.55*	*7*

Selective activations for semantic orienting compared to spatial orienting. All activations were significant at *p* < .05 (corrected) except those shown in italics which were highly significant using a small volume correction based on a 10 mm radius sphere centered on the peak from the first experiment. The number of subjects (*N*) showing this activation at *p* < .01 (uncorrected) is shown in the final column.
